# Acquired Methemoglobinemia Following Herbal Decoction Ingestion Presenting With Refractory Hypoxemia and Associated Generalized Seizure: A Case Report

**DOI:** 10.7759/cureus.111707

**Published:** 2026-06-29

**Authors:** Shivam Bhattad, Fahad Idrees Shaikh, Nishka Tiwari, Vivek Pande, Mausam Jain

**Affiliations:** 1 General Medicine, N.K.P. Salve Institute of Medical Sciences and Research Centre and Lata Mangeshkar Hospital, Nagpur, IND; 2 Medicine, N.K.P. Salve Institute of Medical Sciences and Research Centre and Lata Mangeshkar Hospital, Nagpur, IND; 3 Internal Medicine, N.K.P. Salve Institute of Medical Sciences and Research Centre and Lata Mangeshkar Hospital, Nagpur, IND

**Keywords:** herbal medicine, methemoglobinemia, methylene blue, refractory hypoxemia, saturation gap, seizure

## Abstract

Acquired methemoglobinemia is an uncommon but potentially life-threatening disorder characterized by oxidation of hemoglobin iron into the ferric state, resulting in impaired oxygen delivery and functional anemia. Patients may present with cyanosis, persistent hypoxemia, and discordance between pulse oximetry and arterial oxygen saturation (SaO₂). Although medications and industrial chemicals are well-recognized causes, herbal and traditional remedies remain underreported aetiologies.

A 35-year-old man presented with a sudden-onset generalized tonic-clonic seizure followed by persistent hypoxemia and peripheral cyanosis after ingestion of a home-prepared herbal (Ayurvedic) decoction. The blood sample demonstrated characteristic chocolate-brown discoloration. Oxygen saturation remained approximately 85% despite supplemental oxygen therapy. Arterial blood gas (ABG) analysis demonstrated preserved arterial oxygen tension (PaO₂ 167 mmHg) with a calculated SaO₂ of 97.3%, indicating a characteristic saturation gap. Co-oximetry demonstrated a methemoglobin level of 19%. The diagnosis of methemoglobinemia was supported by refractory hypoxemia, cyanosis, chocolate-brown blood discoloration, saturation gap, and co-oximetry-confirmed methemoglobinemia (19%). The patient improved rapidly following treatment with intravenous methylene blue, anticonvulsants, and supportive care. There was a rapid disappearance of cyanosis; oxygen saturation returned to normal with full neurological recovery.

This example highlights the importance of checking for methemoglobinemia in patients with symptoms of unexplained cyanosis, refractory hypoxemia, and a mismatch between pulse oximetry and arterial oxygen levels, particularly following exposure to herbal or traditional preparations. Recognition of these findings facilitated prompt diagnosis and treatment in our patient.

## Introduction

Methemoglobinemia is a disorder characterized by oxidation of hemoglobin iron from the ferrous (Fe²⁺) to ferric (Fe³⁺) state, resulting in impaired oxygen-carrying capacity and reduced tissue oxygen delivery [1]. Methemoglobin increases the oxygen affinity of the remaining ferrous heme groups, thereby impairing oxygen unloading at the tissue level. Acquired methemoglobinemia is more common than the congenital form and is usually associated with exposure to oxidizing agents such as nitrates, dapsone, benzocaine, sulfonamides, and industrial chemicals [2].

Clinical manifestations vary according to methemoglobin concentration and patient susceptibility, ranging from asymptomatic cyanosis to severe hypoxic injury, metabolic acidosis, neurological impairment, arrhythmias, and death [3]. Cyanosis and refractory hypoxemia are among the most important bedside clues. One of the hallmark diagnostic findings is the presence of a “saturation gap,” wherein pulse oximetry demonstrates persistently low oxygen saturation despite preserved arterial oxygen tension (PaO₂) on arterial blood gas (ABG) analysis [4].

Despite increasing global use of herbal and traditional medicines, their association with methemoglobinemia remains underrecognized [5]. Cases of acquired methemoglobinemia have been reported following exposure to various traditional and herbal preparations containing oxidizing substances capable of inducing hemoglobin oxidation; however, such exposures remain infrequently reported and may be overlooked during clinical evaluation. Increasing use of unregulated traditional medications may contribute to underdiagnosis of toxicological emergencies such as methemoglobinemia.

While cyanosis and refractory hypoxemia are well-recognized manifestations, significant neurological symptoms such as seizures are uncommon and are generally associated with substantially higher methemoglobin concentrations. Furthermore, limited literature exists regarding methemoglobinemia associated with uncharacterized Ayurvedic herbal preparations and the occurrence of neurological manifestations disproportionate to measured methemoglobin levels. The present case is noteworthy because it describes a generalized tonic-clonic seizure following ingestion of a home-prepared Ayurvedic herbal decoction despite a relatively modest methemoglobin level of 19%. This report highlights an important diagnostic challenge and underscores the need to consider methemoglobinemia in patients presenting with unexplained cyanosis, refractory hypoxemia, and neurological manifestations following exposure to herbal or traditional remedies.

## Case presentation

A 35-year-old man presented to the emergency department with sudden-onset involuntary movements involving all four limbs associated with upward rolling of eyeballs and clenching of teeth, followed by vomiting. The patient had been asymptomatic approximately one hour prior to the event.

There was no history of fever, head trauma, focal neurological deficits, chest pain, syncope, bowel or bladder incontinence, tongue bite, rash, or jaundice. The patient reported ingestion of a home-prepared Ayurvedic herbal decoction one day prior to presentation, although the exact constituents were unknown. The chronological sequence of events is summarized in Table 1.

**Table 1:** Timeline of clinical events from herbal decoction ingestion to recovery

Timeline	Clinical Event
Day −1	Ingestion of the Ayurvedic herbal decoction
Day 0	First generalized tonic-clonic seizure
Day 0	Persistent hypoxemia detected (SpO_2_≈85%)
Day 0	Peripheral cyanosis was identified, and chocolate-brown blood was noted
Day 0	Saturation gap observed on arterial blood gas
Day 0	Clinical suspicion of methemoglobinemia raised
Day 0	Intravenous methylene blue was administered
Day 1	Resolution of cyanosis and normalization of oxygen saturation
Day 2	Initial co-oximetry revealed methemoglobinemia with a methemoglobin fraction of 19%. Repeat co-oximetry after methylene blue administration showed a reduction in methemoglobin levels to approximately 6%, accompanied by resolution of cyanosis and normalization of oxygen saturation.
2-week follow-up	No recurrent seizures or hypoxic episodes

There was no known history of seizure disorder, diabetes mellitus, hypertension, pulmonary disease, or cardiac illness. The patient was a chronic tobacco chewer.

On examination, the patient was drowsy but arousable to deep, painful stimuli. Pulse rate was 110 beats/minute, blood pressure was 100/70 mmHg, respiratory rate was 20 breaths/minute, and oxygen saturation was 85% on room air. Oxygen saturation remained refractory to supplemental oxygen therapy via face mask. Peripheral cyanosis with bluish discoloration of the tongue and nail beds was noted. The patient's blood appeared characteristically dark brown (chocolate brown) in color, raising suspicion of dyshemoglobinemia. Respiratory, cardiovascular, abdominal, and neurological examinations were otherwise unremarkable. During evaluation, the patient developed another episode of generalized tonic-clonic seizure. The MRI of the brain was unremarkable. Electroencephalography (EEG) and toxicological analysis of the ingested herbal preparation were not available during the patient's evaluation. There was no prior history of epilepsy, head trauma, fever, alcohol withdrawal, or known neurological disease.

ABG analysis while receiving supplemental oxygen was as follows: pH: 7.327; PaO₂: 167 mmHg; arterial oxygen saturation (SaO₂): 97.3%; bicarbonate (HCO₃⁻): 20.3 mmol/L.

Laboratory and ABG findings are summarized in Table 2.

**Table 2:** Laboratory and arterial blood gas investigations

Investigation	Result	Reference Range
pH	7.327	7.35–7.45
Partial pressure of arterial oxygen ( PaO₂)	167 mmHg	80–100 mmHg
Arterial oxygen saturation (SaO₂)	97.3%	>95%
Bicarbonate (HCO₃⁻)	20.3 mmol/L	22–26 mmol/L
Co-oximetry (Methemoglobin)	19%	<1.5%
Co-oximetry (Repeat sample)	6%	<1.5%
Liver enzymes	Mild elevation	—
Random blood sugar	156 mg/dL	70–140 mg/dL
Serum sodium	138 mEq/L	135–145 mEq/L
Serum potassium	4.22 mEq/L	3.5–5.0 mEq/L
Serum calcium	8.50 mg/dL	8.5–10.5 mg/dL

The preserved PaO₂ and low pulse oximetry readings indicated that dissolved oxygen content remained normal despite impaired oxygen-carrying capacity. This finding reflects the distinction between PaO₂, which measures dissolved oxygen in plasma, and functional oxygen saturation, which depends on the proportion of hemoglobin capable of binding and transporting oxygen. In methemoglobinemia, PaO₂ may remain normal or elevated because pulmonary oxygen transfer is preserved; however, oxidation of hemoglobin to the ferric state reduces the fraction of hemoglobin available for oxygen transport, resulting in impaired tissue oxygen delivery despite adequate PaO₂. Pulse oximetry continued to show oxygen saturation levels of only about 85%, even though PaO₂ was normal, indicative of a saturation gap. The discrepancy between pulse oximetry and arterial oxygenation is demonstrated in Table 3.

**Table 3:** Demonstration of saturation gap between pulse oximetry and arterial oxygenation The discrepancy between low SpO₂ and preserved SaO₂ with normal-to-elevated PaO₂ represents a characteristic “saturation gap,” an important diagnostic clue suggestive of methemoglobinemia and other dyshemoglobinemias.

Parameter	Value
Pulse oximetry saturation (SpO₂)	85%
Calculated arterial oxygen saturation (SaO₂)	97.3%
Partial pressure of arterial oxygen ( PaO₂)	167 mmHg

In addition, the blood sample demonstrated characteristic chocolate-brown discoloration, further raising suspicion for methemoglobinemia. Co-oximetry demonstrated a methemoglobin level of 19%. Routine laboratory investigations, including complete blood count, renal function tests, serum electrolytes, glucose-6-phosphate dehydrogenase (G6PD) activity, chest radiography, electrocardiography (ECG), abdominal ultrasonography, and transthoracic echocardiography (TTE), were unremarkable except for mild transaminitis.

The patient was treated with high-flow supplemental oxygen and intravenous methylene blue administered at approximately 1.5 mg/kg diluted in 5% dextrose. Anticonvulsant therapy with levetiracetam and supportive care were also given.

Normal G6PD activity allowed safe administration of methylene blue. Following methylene blue administration, rapid improvement was seen in cyanosis and oxygen saturation. Repeat co-oximetry demonstrated a reduction of the methemoglobin level to 6%. No further seizures occurred during hospitalization. The patient recovered completely and was discharged with an oxygen saturation of 98% on room air. At the two-week follow-up, he remained asymptomatic without recurrence of seizures or hypoxic symptoms.

## Discussion

Methemoglobinemia is a crucial, although frequently overlooked, cause of refractory hypoxemia. When hemoglobin iron gets oxidized from Fe²⁺ to Fe³⁺ state, besides impairing oxygen transport, it also causes the oxygen-hemoglobin dissociation curve to shift from right to left, decreasing the oxygen delivery to the tissues and causing functional anemia [6]. In fact, one of the most important diagnostic clues in this case was the presence of a saturation gap. Pulse oximetry consistently showed oxygen saturation around 85%, whereas ABG analysis revealed preserved PaO₂ and a calculated oxygen saturation of 97.3%. Co-oximetry remains the diagnostic gold standard for methemoglobinemia because conventional pulse oximeters use limited wavelengths and are unable to reliably differentiate dyshemoglobins from normal hemoglobin species. Consequently, pulse oximetry may underestimate oxygen saturation despite preserved arterial oxygenation [6]. The sustained discordance between pulse oximetry and arterial oxygenation strongly points to dyshaemoglobinemia and, in fact, is a classical manifestation of methemoglobinemia [7]. Most pulse oximeters are designed with very few wavelengths, and due to this factor, they are incapable of differentiating dyshemoglobin species from normal hemoglobin species. This leads to the situation of pulse oximeters showing incorrect low oxygen saturation levels even when the arterial oxygen content is sufficiently high. Cyanosis, along with a normal or high PaO₂, should highlight the possibility of dyshemoglobinemias, mainly methemoglobinemia, among other considerations [8].

Another major bedside sign in this case was the characteristic chocolate-brown discoloration of blood, which is usually the result of the iron in the hemoglobin getting oxidized to the Fe³⁺ state. The combination of refractory hypoxemia, peripheral cyanosis, chocolate-brown blood discoloration, saturation gap, and elevated methemoglobin level strongly supported the diagnosis of methemoglobinemia. The diagnosis was initially challenging because the persistent hypoxemia raised concern for underlying cardiopulmonary pathology, even though arterial oxygenation was preserved. Pulmonary embolism, pneumonia, intracardiac shunt, and other cardiopulmonary disorders were also considered. But the normal imaging studies, the unremarkable cardiovascular evaluation, and the very typical saturation gap made these diagnoses very unlikely (Table 4), and methemoglobinemia was preferred [9]. Other reasons for the acute symptomatic seizure were discussed as well. Neuroimaging was normal, and the patient did not have epilepsy, head trauma, fever, alcohol withdrawal, or any known neurological disease. Laboratory routine investigations did not point to any other metabolic causes either. Random blood glucose was 156 mg/dL, serum sodium was 138 mEq/L, serum potassium was 4.22 mEq/L, and serum calcium was 8.50 mg/dL, making hypoglycemia and major electrolyte disturbances unlikely causes of the seizure episode. Nevertheless, since the toxicological characterization of the ingested herbal decoction preparation was unavailable, the possibility of an unidentified neurotoxic component contributing to seizure activity cannot be entirely excluded.

**Table 4:** Differential diagnosis of refractory hypoxemia and the reasons for exclusion

Differential Diagnosis	Reason Excluded
Pulmonary embolism	Preserved arterial oxygenation, absence of suggestive imaging findings, and rapid response to methylene blue made pulmonary embolism unlikely
Pneumonia	No fever, respiratory symptoms, or radiographic evidence of pulmonary infection
Cardiac shunt	Echocardiography was unremarkable, and arterial oxygenation remained preserved.
Primary seizure disorder	No prior history of epilepsy; neuroimaging was normal, and no recurrent seizures occurred following correction of methemoglobinemia
Metabolic seizure	Routine biochemical investigations were unremarkable.
Toxic ingestion	Considered; specific toxicological analysis unavailable
Carbon monoxide poisoning	Clinical presentation and arterial blood gas findings were inconsistent.
Methemoglobinemia	Saturation gap, cyanosis, chocolate-brown blood discoloration, elevated methemoglobin level, and response to methylene blue

Usually, neurological symptoms are seen at very high levels of methemoglobin, commonly >20%-30% [10]. Although seizures have been reported in severe methemoglobinemia, they are typically associated with substantially higher methemoglobin concentrations. The occurrence of generalized tonic-clonic seizure at a measured methemoglobin level of 19% in the present case is, therefore, unusual and suggests that factors beyond the measured methemoglobin concentration may have contributed to neurological dysfunction. Potential explanations include transient periods of greater methemoglobinemia prior to presentation, individual susceptibility to tissue hypoxia, or exposure to unidentified neurotoxic constituents within the ingested herbal preparation. Another reason for the seizure could be that it was not only caused by the oxidized hemoglobin but also by other components of the herbal preparation that have not yet been identified. The possibility that unidentified constituents of the ingested herbal preparation contributed to seizure activity cannot be completely excluded. This case highlights the challenges of establishing direct clinic-biochemical correlation in patients exposed to uncharacterized toxic substances [10].

Herbal and traditional medicines are still overlooked causes of acquired methemoglobinemia. The preparations that are not regulated can have oxidizing agents that might lead to the oxidation of hemoglobin, causing the formation of methemoglobin [11]. A temporal association was observed between ingestion of the Ayurvedic herbal decoction and the subsequent development of methemoglobinemia. However, because the toxicological characterization of the preparation was unavailable, a definitive causal relationship cannot be established. Besides, the mild increase of liver enzymes in this patient might be due to a short period of hypoxia caused by the tissue or simply the direct toxic effect of the preparation that was ingested [11].

The diagnosis was further strengthened by the patient's rapid clinical improvement following intravenous methylene blue administration. Methylene blue facilitates the reduction of ferric iron to the ferrous state via the NADPH-dependent methemoglobin reductase pathway so that the hemoglobin goes back to being able to carry oxygen [12]. Resolution of cyanosis, normalization of oxygen saturation, reduction in methemoglobin level, and absence of recurrent neurological symptoms following treatment provided additional support for the diagnosis of acquired methemoglobinemia [12]. A comparison of selected published cases of acquired methemoglobinemia with the present case is summarized in Table 5.

**Table 5:** Clinical characteristics and outcomes of selected published cases of acquired methemoglobinemia

Author (Reference)	Etiology	Clinical Presentation	Diagnostic Clue	Treatment	Outcome
Tazón-Varela MA et al. [5]	Oxidizing inhalational exposure	Cyanosis and refractory hypoxemia	Saturation gap with low peripheral oxygen saturation	Intravenous methylene blue	Recovery
Thiri T et al. [8]	Acquired methemoglobinemia	Cyanosis, hypoxemia, altered sensorium	Preserved partial pressure of arterial oxygen with low pulse oximetry	Methylene blue and oxygen therapy	Recovery
Bhatta S and Awasthi PR [9]	Drug/toxin-induced methemoglobinemia	Refractory hypoxemia and cyanosis	Co-oximetry confirmation	Methylene blue	Recovery
Present case	Ayurvedic kadha	Refractory hypoxemia, cyanosis, and associated generalized tonic-clonic seizure	Saturation gap with elevated methemoglobin	Intravenous methylene blue	Complete recovery

The exact composition of the consumed herbal preparation could not be determined because toxicological analysis of the preparation was unavailable. Furthermore, EEG and comprehensive toxicological screening were not performed, limiting definitive attribution of the seizure solely to methemoglobinemia. Although the clinical findings and therapeutic response strongly supported the diagnosis of methemoglobinemia, a contribution from unidentified neurotoxic constituents within the herbal preparation cannot be completely excluded. Although the diagnosis of methemoglobinemia was supported by the presence of a saturation gap, chocolate-brown blood discoloration, co-oximetry-confirmed methemoglobinemia (19%), and a prompt clinical response to methylene blue, a contribution from unidentified toxic constituents within the herbal preparation cannot be completely excluded. Serial toxicological evaluation and characterization of the ingested preparation were not available. Repeat co-oximetry following methylene blue administration demonstrated a reduction in methemoglobin level to approximately 6%; however, additional serial measurements were not available to further characterize the kinetics of methemoglobin clearance.

## Conclusions

Acquired methemoglobinemia should be considered in patients presenting with refractory hypoxemia, peripheral cyanosis, chocolate-brown blood discoloration, and discrepancy between pulse oximetry and arterial oxygenation. Recognition of a saturation gap can facilitate early bedside diagnosis and help avoid unnecessary cardiopulmonary investigations. This case highlights the value of early co-oximetry and careful exposure history-taking, including inquiry regarding the use of herbal and traditional remedies. Although a temporal association was observed between ingestion of a home-prepared herbal decoction and the development of methemoglobinemia, a definitive causal relationship could not be established because toxicological characterization of the preparation was unavailable. Similarly, while generalized tonic-clonic seizure occurred in conjunction with methemoglobinemia, the contribution of other unidentified toxic constituents cannot be completely excluded. Prompt recognition of characteristic clinical findings and timely administration of methylene blue remain essential for favorable outcomes.
